# The Effects of Oral Magnesium Supplementation on Glycemic Response among Type 2 Diabetes Patients

**DOI:** 10.3390/nu11010044

**Published:** 2018-12-26

**Authors:** Wafaa A. ELDerawi, Ihab A. Naser, Mahmmoud H. Taleb, Ayman S. Abutair

**Affiliations:** Department of Clinical Nutrition, Faculty of Applied Medical Sciences, Al-Azhar University-Gaza, Jamal Abdl Naser St., P.O. Box 1277, Gaza, Gaza Strip 00970, Palestine; wafaa@mecaforpeace.org (W.A.E.); ihabnaser@yahoo.ca (I.A.N.); mahtaleb@hotmail.com (M.H.T.)

**Keywords:** diabetes mellitus, magnesium, blood glucose, insulin

## Abstract

Background: Magnesium (Mg) supplementation may help control glycemic response among type 2 diabetes (T2D) patients. Objective: This study means to determine whether Mg supplementation improves glycemic control indicators in patients with T2D. Methods: After one week of the dietary stabilization phase, 42 T2D patients were stratified according to sex, age, fasting blood sugar (FBS) and Mg levels and then randomly allocated into two groups. The intervention group was on 250 mg/day of elemental Mg for three months while the control group did not receive any type of supplements throughout the intervention period. Results: The daily administration of 250 mg of elemental Mg indicated a significant improvement in HbA1C (8.32 to 7.96%, *p* < 0.001), insulin levels (IL) (15.56 to 12.18 μIU/mL, *p* < 0.001), C-peptide (2.28 to 1.90 ng/mL, *p* = 0.001), HOMA.IR (6.16 to 4.44, *p* < 0.001) and HOMA.β% (59.99 to 52.37, *p* = 0.036) of the intervention group when compared with the control group after three months of intervention. Conclusion: The results of this study revealed that oral Mg supplementation reduces insulin resistance and improves the glycemic control indicators among T2D patients. Trial registration: current controlled trials PHRC/HC/32/15. Registered 5 October 2015.

## 1. Background

Type 2 diabetes (T2D) is a chronic metabolic disorder resulting from defects in insulin secretion, insulin action or both, leading to hyperglycemia [1]. Chronic hyperglycemia leads to heart disease, stroke, kidney disease, blindness and amputation [2]. According to the World Health Organization, the prevalence of diabetes is increasing at an alarming rate; 422 million people worldwide have diabetes [3]. Several studies were conducted to improve the glycemic control indicators in T2D patients. They used different types of supplements, such as vitamin D [4], vitamin C [5], and dietary fibers [6], which have proven to be effective as potential diabetes risk modifiers. Furthermore, Mg supplements have been suggested to be an adjuvant therapy in the prevention and management of diabetes [7,8].

Poor intracellular Mg concentration and increased intracellular free calcium, as found in T2D patients, may cause insulin resistance [9]. In contrast, higher Mg levels corresponded to a greater degree of sensitivity to insulin [10]. The importance of Mg on insulin sensitivity was suggested in the early 1980s [11] and resulted in the following clinical evidence. Some studies reported the beneficial effects of Mg supplementation on metabolic control in individuals with T2D [8,12,13,14] while, at the same time, other studies showed no significant effects of Mg supplementation on T2D [15,16]. Hence, the effect of Mg supplementation in T2D remained controversial in literature. This study was one of the first to describe the impacts of Mg supplementation among T2D patients in the Gaza Strip. The aim of this study was to investigate the effects of adding Mg supplementation to the normal daily diet of T2D patients for three months of intervention.

## 2. Subjects and Methods

In this randomized, controlled trial we included 64 patients who were newly diagnosed (maximum one year) with T2D and who were available and willing to participate. Therefore, patients with T2D aged 35–60 years old where recruited from private endocrine clinics. Before the study, all the patients were interviewed and clinically evaluated; blood samples were drawn in order to verify their eligibility criteria. Forty-eight patients were found to be eligible to enter the one-week dietary stabilization phase. During this phase, six patients were unable to comply with the general guidelines of the dietary stabilization phase and were excluded. The remaining 42 T2D patients were ultimately enrolled in the study and stratified according to sex, age, fasting blood sugar (FBS) and Mg levels. The targeted subjects were randomly allocated into two groups (Mg group and control group). Only two cases failed to complete the intervention period.

### 2.1. Declarations: Ethics Approval and Consent to Participate

The present study was approved by the Helsinki Committee (PHRC/HC/32/15), the Deanship of the Faculty of Pharmacy, Graduate Studies and the Union of Health Work Committees. Consent was obtained from the participants.

### 2.2. Consent for Publication

Written informed consent for publication was obtained from the participants.

### 2.3. Eligibility Criteria

Both genders were included in this study. Participants aged 35–60 years with the FBS of more than 126 mg/dL who had been recently diagnosed with T2D (maximum one year) and were being treated with antidiabetic drugs were asked to participate. Those who agreed to participate in the trial were asked not to make any changes to their lifestyle or dietary pattern during the intervention program. Criteria for exclusion from the study included: Patients who used insulin, calcium channel blockers agents, Mg, calcium containing supplements, and/or diuretic drugs. Other factors for exclusion from the study were reduced renal function (serum creatinine levels of more than 1.3 mg/dL in women and more than 1.5 mg/dL in men), elevated hepatic enzymes (more than three fold over normal values), recent infections (less than one month prior to study), chronic inflammatory diseases, cerebrovascular accidents, acute coronary syndrome (less than one month prior to the study), pregnancy or lactation, chronic diarrhea, or participation in other clinical trials.

### 2.4. Study Design

The present study employed a randomized clinical trial design to determine whether Mg supplementation improves glycemic control indicators in T2D patients.

### 2.5. Intervention Protocol

During the dietary stabilizing phase, all subjects were instructed to follow a prescribed diet plan for one week to stabilize their serum glucose level (30% of total energy as fat, 15% of energy as protein, and 55% of energy as carbohydrate-focused, from complex carbohydrates).

The intervention group consisted of 20 participants who were on Mg supplementation for three months. The control group (20 participants) did not receive any type of supplement throughout the intervention period. Both groups were instructed to consume a healthy diet of fruits and vegetables (five serving per day), legumes, nuts and whole grains. Subjects were instructed to consume less than 5% of total energy intake from free sugars, which is equivalent to 25 g for a person of health body mass consuming approximately 2000 Kcal/day, and less than 30% of total energy intake from fats. Saturated fats (e.g., found in fatty meat, butter, palm and coconut oil, cream, cheese, ghee and lard) were to be replaced by unsaturated fats (e.g., found in fish, avocado, nuts, sunflower, canola and olive oils). Less than 5 g of salt per day, the equivalent of one teaspoon, was prescribed.

Jamieson magnesium tablets were used, each containing 250 mg of elemental high-potency, highly absorbable magnesium (oxide, gluconate, lactate). The supplements contained no salt, sugar, starch, gluten, or lactose. Each patient in the intervention group used one Mg tablet per day for 3 months.

To ensure patients adhered to the intervention program, all patients of both groups were, when possible, met with weekly and contacted by phone twice a week. If a meeting was not possible they were contacted only by phone. In order to evaluate the compliance of the respondents, the subjects’ checklists containing the number of days and number of tablets consumed were examined weekly and the remaining tablets counted to learn of any missed dose.

### 2.6. Measurements

All measurements and indicators were taken at the base line after three months of intervention. Body weight and height were measured to calculate body mass index (BMI). Blood samples were collected into centrifuge tubes. Blood was allowed to clot at room temperature for about 1 h and then centrifuged at 3000 rpm for 10 min. The serum was carefully separated into storage tubes and frozen at −20 °C prior to analysis for biochemical tests.

### 2.7. Biochemical Analysis

The biochemical parameters included: FBS, serum Ca, serum Mg, HbA1c, fasting C-peptide levels, and fasting insulin levels. FBS was measured using the glucose oxidase method (GOD-PAP kit), where the normal level in the plasma is 70–115 mg/dL. Total cholesterol (TC) was measured using a CHOD-POD cholesterol kit. The recommended reference value was less than 200 mg/dL, with 200–239 mg/dL as the upper limit, and more than 240 mg/dL being high. Triglycerides (TGs) were measured using a triglyceride GPO-POD kit and the desirable reference value for TGs was less than 150 mg/dL, with 150–200 as the upper limit and more than 200 mg/dL being high. High density lipoprotein (HDL) was measured using a liquid HDL precipitant kit and the reference values for heart disease were as follows: Less than 40 mg/dL high risk, 40–59 mg/dL moderate risk, more than 60 mg/dL low risk. Serum Ca was measured using a photometric test (Cresolphthalein-complexone kit) where the normal range of total Ca in the serum was 8.1–10.4 mg/dL. Serum Mg was measured using the colorimetric method (Calmagite kit) where the normal range is 1.6–3 mg/dL. Alanine aminotransferase (ALT) was measured using the photometric method (LDH-NADH kinetic UV liquid), for which the normal level for females is less than 32 U/L and for males is less than 40 U/L. The device used for HbA1c analysis was a Clover A1c analyzer from Belgium and the kit used was a Clover A1c test cartridge. The device used for insulin and C-peptide analysis was a SNIBE CLIA analyzer from China where MAGLUMI Insulin CLIA kits and VAST ELISA micro-wells systems are used for measuring IL and C-peptide, respectively.

The HOMA-IR and HOMA.β% were calculated according to the following formulas: HOMA-IR = (glucose mg x insulin level)/405, and HOMA.β% = [(360 × insulin level)/(glucose (mg/dL − 63)] [17]. The HOMA model is calculated in order to predict insulin sensitivity and β-cell function from fasting plasma insulin and glucose concentrations. The relationship between glucose and insulin in the basal state reflects the balance between hepatic glucose output and insulin secretion. The optimal range of HOMA-IR is 1.0 × (0.5–1.4). A HOMA-IR less than 1.0 indicates that the human body is sensitive to insulin, above 1.9 indicates early insulin resistance, and above 2.9 indicates significant insulin resistance. A lower HOMA.β% value indicates greater β-cell dysfunction.

### 2.8. Data Management and Analysis

The data was entered into an SPSS (statistical package for social sciences) version 20.0 database for Windows. Data treatment included data cleaning and defining and recoding certain variables. The data analysis was divided into two steps: Descriptive statistics and analysis of variance. The quantitative data were represented in the form of proportions (%) and of means with standard deviations. The one-way repeated measure ANOVA was used to measure the changes between groups.

## 3. Results

Forty respondents successfully completed the trial from beginning to end. The primary data (sex, age, BMI, FBS, and Mg) were statistically analyzed and there were no significant differences between the groups at the base-line level. Hence, no further adjustment was required.

Table 1 shows that there were no statistically significant differences in any base-line characteristics between the groups.

Table 2 presents the changes in the glycemic control indicators in both groups after three months of intervention. The HbA1c, insulin levels, C-peptide, HOMA.IR, and HOMA.β% improved significantly (0.36% *p* < 0.001, 3.47 μIU/mL *p* < 0.001, 0.38 ng/mL *p* = 0.001, 1.72 *p* < 0.001, 7.628 *p* = 0.036, respectively) in the intervention group when compared with the control group after three months of intervention. In addition, there were no statistical differences between the FBS of the groups but, with reference to mean differences, the FBS was reduced by 10.55 mg/dL in the intervention group and, unfortunately, increased in the control group by 10.1 mg/dL.

Table 3 shows that Ca levels and the Ca/Mg ratio significantly decreased (0.575 mg/dL, *p* = 0.001 and 0.435, *p* = 0.001, respectively). On the other hand, the Mg level increased significantly (0.065 mg/dL, *p* = 0.036) in the intervention group when compared with the control group after three months of intervention.

## 4. Discussion

The results of the intervention group showed a significant reduction in serum calcium levels (8.98 to 8.40 mg/dL) while there was a significant increase in Mg levels (1.99 to 2.06 mg/dL). These results led to a significant reduction in the Ca/Mg ratio (4.50 to 4.11). These results reflect the role of Mg as a mild Ca antagonist [18] which could be explained by the similarities between Ca and Mg in chemical reactivity and charge [19]. Mg and Ca antagonize each other in re-absorption, inflammation and many other physiological activities. The absorbed amount of Ca or Mg depended on the dietary ratio of Ca to Mg intake [20]. These findings were in line with previously reported findings which indicated that Mg salts dissolve easily in water and are much more soluble than the respective calcium salts. As a result, Mg is readily available to organisms [21].

The results also coincided with Moran and his colleague’s trial which reported that the administration of a 50 mL MgCl_2_ solution for 16 weeks significantly increased serum Mg concentration compared with a placebo [12]. In contrast, another clinical trial showed non-significant differences in the serum Mg and Ca/Mg ratio after administration of 300 mg Mg supplements for three months [8].

After three months of intervention, significant reductions were observed in the plasma levels of HbA1c, insulin levels, C-peptide, and HOMA-IR and HOMA.β% (*p* < 0.001, *p* < 0.001, *p* = 0.001, *p* < 0.001, *p* = 0.036, respectively). Though the results of the intervention group showed marked reduction in FBS (158.6 to 148.05 mg/dL), these improvements in the FBS were marginally insignificant when compared with the control group. The insignificant results between the groups regarding FBS might be attributed to a lack of adherence to the prescribed diet in the days before measurements. The results reported significant reduction in FBS (*p* = 0.009) in pre-post change in comparison with the intervention group.

Solati and his coworkers did not report any significant improvement in the Mg levels, though there was a significant improvement in the FBS (183.9 to 125.8 mg/dL) and the 2 h postprandial blood glucose (239.1 to 189.1 mg/dL) [8]. Interestingly, our intervention group reported significant improvement in Mg levels as well as in FBS and other glycemic control indicators. This might be attributed to: (1) the dietary stabilization phase, (2) the prescribed diet, and (3) the strict implementation of monitoring procedures. Guerrero-Romero reported that the intake of 50 mL MgCl2 for 16 weeks significantly improved HOMA-IR, FBS, and HbA1c in T2D patients [22].

Higher Mg levels corresponded to a greater degree of sensitivity to insulin [10] and this explained the improvement in the glycemic control indicators after Mg supplementation. On the other hand, the improvement could be explained by different mechanisms including the influence of Mg on insulin receptor activity through enhanced tyrosine kinase phosphorylation [23,24,25]. With regard to the role of Mg as a mild Ca antagonist which inhibits calcium-induced cell death, increases in intracellular Ca may play a pathogenic role in insulin resistance syndrome and trigger cell death. These improvements in the glycemic indicators were not only related to the improvement in insulin sensitivity, there was the possibility that Mg could help in facilitating the translocation of glucose transporter number 4 (GLUT 4) to the cell membrane. This would take place by the activation of tyrosine–kinase in the presence of Mg [9].

The results of this study matched previous studies that concluded that daily oral Mg supplementation substantially improved insulin sensitivity by 10% and reduced blood sugar by 37% [12,22]. The results also agreed with Chacko et al., who examined the effects of oral Mg supplementation (500 mg elemental Mg/d for four weeks) on metabolic biomarkers in overweight individuals and reported that Mg treatment significantly improved fasting C-peptide concentrations and appeared to improve fasting insulin concentrations [14]. Moreover, the results are in accordance with the Mooren clinical trial, which reported significant improvements in FBS and some insulin sensitivity indices compared with the placebo after six months of Mg supplementation [26]. Meanwhile, Yokota and co-workers, in their clinical trial, reported a significant reduction in insulin levels and HOMA-IR, but not in FBS and HbA1c after Mg supplementation [27]. The differences in the results of the previous clinical trials, which investigated the effectiveness of Mg supplements on glycemic control indicators, could be explained by the differences in the Mg dosages and the duration of supplementation.

## 5. Conclusions

This study investigated one of the controversial points in the strategies of prevention and management of diabetes. According to the results, oral Mg supplementation significantly improved HbA1C, IL, C-peptide, HOMA.IR and HOMA.β% and insignificantly decreased FBS. In addition, the use of Mg supplements reduced insulin resistance and improved glycemic control indicators among T2D patients.

## 6. Availability of Data and Materials

According to the policy of BMC, we would like to inform you that all the datasets analyzed during the current study are available from the corresponding author upon reasonable request.

## Figures and Tables

**Table 1:** Baseline characteristics of the respondents.

Variables	Control Group (*n* = 20)	Intervention Group (*n* = 20)	*p* Value
Age	51.55 (8.287)	51.15 (7.005)	0.870
Sex			
Male	11	10	
Female	9	10	
BMI	30.00 (4.56)	29.02 (5.07)	0.745
FBS (mg/dL)	161.00 (25.25)	158.50 (43.75)	0.862
HbA1c (%)	8.15 (1.88)	7.90 (0.95)	0.547
IL (μIU/mL)	15.61 (7.52)	11.92 (5.87)	0.086
C-peptide (ng/mL)	2.15 (1.28)	2.00 (0.68)	0.583
HOMA.IR	5.58 (2.81)	4.70 (3.32)	0.127
HOMA.β%	61.24 (28.99)	51.23 (36.38)	0.277
Ca (mg/dL)	9.00 (0.4)	8.85 (0.57)	0.602
Mg (mg/dL)	1.95 (0.38)	2.00 (0.28)	0.369
Ca/Mg ratio	4.66 (0.85)	4.49 (0.8)	0.289

The level of significant is <0.05.

**Table 2:** Differences in the glycemic control indicators between the groups.

Variables	Control (*n* = 20)		Intervention (*n* = 20)		*p* Value ^a^
	Pre ^b^	Post	Pre	Post	
FBS (mg/dL)	159.05(30.83)	168.6(31.36)	158.6(15.06)	148.05(10.52) **	0.068
HbA1c (%)	8.02 (0.836)	8.49 (0.959) **	8.32(1.239)	7.96(1.028) **	*p* < 0.001
IL (μIU/mL)	13.06 (4.565)	16.75(5.66) **	15.65(4.556)	12.18(2.806) **	*p* < 0.001
C-peptide (ng/mL)	2.19(0.667)	2.49 (0.703) *	2.28 (0.716)	1.90 (0.336) *	0.001
HOMA.IR	5.14 (2.048)	7.15 (3.347) **	6.16 (2.08)	4.44 (1.06) ***	*p* < 0.001
HOMA.β%	53.24 (21.884)	59.21 (20.037)	59.99 (17.9)	52.37 (13.363) *	0.036

^a^ is the repeated measure ANOVA between the control and intervention groups; ^b^ is the mean-standard deviation; <0.05 is the level of significance; * demonstrates significant differences in the Wilcoxon test between the base-line and the three months in the same group, * *p* < 0.05, ** *p* < 0.01, *** *p* < 0.001.

**Table 3:** Differences in the Ca, Mg, and Ca/Mg ratio between the groups.

Variables	Control (*n* = 20)		Intervention (*n* = 20)		*p* Value ^a^
	Pre ^b^	Post	Pre	Post	
Ca (mg/dL)	8.94 (0.45)	8.91(0.579)	8.98(0.383)	8.40 (0.246) ***	0.001
Mg (mg/dL)	2.05 (0.212)	1.92 (0.191)	1.99 (0.209)	2.06 (0.19)	0.036
Ca/Mg ratio	4.40 (0.491)	4.68 (0.535)	4.54 (0.489)	4.11 (0.372) **	0.001

^a^ Repeated Measure ANOVA between control and intervention groups; ^b^ Mean (Standard Deviation). The level of significant is <0.05. Asterisk = significantly different by Wilcoxon test between baseline and 3rd months in the same group, ** *p* < 0.01, *** *p* < 0.001.
